# *Betula pendula* Leaf Extract Targets the Interplay between Brain Oxidative Stress, Inflammation, and NFkB Pathways in Amyloid Aβ_1-42_-Treated Rats

**DOI:** 10.3390/antiox12122110

**Published:** 2023-12-13

**Authors:** Alexandra-Cristina Sevastre-Berghian, Irina Ielciu, Timea Bab, Neli-Kinga Olah, Vlad Sever Neculicioiu, Vlad Alexandru Toma, Bogdan Sevastre, Teodora Mocan, Daniela Hanganu, Andreea Elena Bodoki, Ioana Roman, Roxana Liana Lucaciu, Adriana Corina Hangan, Alina-Diana Hașaș, Roxana Maria Decea, Ioana Băldea

**Affiliations:** 1 Department of Physiology, Faculty of Medicine, “Iuliu Hațieganu” University of Medicine and Pharmacy, 400006 Cluj-Napoca, Romania; alexandra_berghian@yahoo.com (A.-C.S.-B.); teodora_mocan@yahoo.com (T.M.); roxanadecea@yahoo.com (R.M.D.); baldeaioana@gmail.com (I.B.); 2 Department of Pharmaceutical Botany, Faculty of Pharmacy, “Iuliu Haţieganu” University of Medicine and Pharmacy, 400337 Cluj-Napoca, Romania; irina.ielciu@umfcluj.ro; 3 PlantExtrakt Ltd., Rădaia, 407059 Cluj-Napoca, Romania; timea.bab@plantextrakt.ro (T.B.); neli.olah@plantextrakt.ro (N.-K.O.); 4 Department of Pharmacognosy, Faculty of Pharmacy, “Iuliu Haţieganu” University of Medicine and Pharmacy, 400010 Cluj-Napoca, Romania; dhanganu@umfcluj.ro; 5 Department of Pharmaceutical Chemistry, Faculty of Pharmacy, “Vasile Goldiş” Western University of Arad, 310025 Arad, Romania; 6 Department of Microbiology, “Iuliu Hatieganu” University of Medicine and Pharmacy, 400349 Cluj-Napoca, Romania; 7 Department of Molecular Biology and Biotechnology, Babes-Bolyai University, 400371 Cluj-Napoca, Romania; 8 Department of Clinical and Paraclinical Sciences, University of Agricultural Sciences and Veterinary Medicine, 400372 Cluj-Napoca, Romania; bogdan.sevastre@usamvcluj.ro (B.S.); alina-diana.hasas@student.usamvcluj.ro (A.-D.H.); 9 Department of Inorganic Chemistry, “Iuliu Haţieganu” University of Medicine and Pharmacy, 400012 Cluj-Napoca, Romania; abota@umfcluj.ro (A.E.B.); acomsa6@yahoo.com (A.C.H.); 10 Department of Experimental Biology and Biochemistry, Institute of Biological Research, 400015 Cluj-Napoca, Romania; ioana.roman@icbcluj.ro; 11 Department of Pharmaceutical Biochemistry and Clinical Laboratory, Faculty of Pharmacy, “Iuliu Hațieganu” University of Medicine and Pharmacy, 8 Victor Babeș Street, 400000 Cluj-Napoca, Romania; roxanaluc@yahoo.com

**Keywords:** brain, oxidative stress, inflammation, NFkB, Aβ_1-42_, *B. pendula* leaf extract

## Abstract

Alzheimer’s disease (AD) is known as the primary and most common cause of dementia in the middle-aged and elderly population worldwide. Chemical analyses of *B. pendula* leaf extract (BPE), performed using spectrophotometric and chromatographic methods (LC/MS), revealed high amounts of polyphenol carboxylic acids (gallic, chlorogenic, caffeic, trans-p-coumaric, ferulic, and salicylic acids), as well as flavonoids (apigenin, luteolin, luteolin-7-O-glucoside, naringenin, hyperoside, quercetin, and quercitrin). Four groups of Wistar rats were used in this experiment (*n* = 7/group): control (untreated), Aβ_1-42_ (2 μg/rat intracerebroventricular (i.c.v.), Aβ_1-42_ + BPE (200 mg/Kg b.w.), and DMSO (10 μL/rat). On the first day, one dose of Aβ_1-42_ was intracerebroventricularly administered to animals in groups 2 and 3. Subsequently, BPE was orally administered for the next 15 days to group 3. On the 16th day, behavioral tests were performed. Biomarkers of brain oxidative stress Malondialdehyde (MDA), (Peroxidase (PRx), Catalase (CAT), and Superoxid dismutase (SOD) and inflammation (cytokines: tumor necrosis factor -α (TNF-α), Interleukin 1β (IL-1β), and cyclooxygenase-2 (COX 2)) in plasma and hippocampus homogenates were assessed. Various protein expressions (Phospho-Tau (Ser404) (pTau Ser 404), Phospho-Tau (Ser396) (pTau Ser 396), synaptophysin, and the Nuclear factor kappa B (NFkB) signaling pathway) were analyzed using Western blot and immunohistochemistry in the hippocampus. The results show that BPE diminished lipid peroxidation and neuroinflammation, modulated specific protein expression, enhanced the antioxidant capacity, and improved spontaneous alternation behavior, suggesting that it has beneficial effects in AD.

## 1. Introduction

Alzheimer’s disease (AD), known as a progressive neurodegenerative disorder, described in 1906, and named after the German psychiatrist Alois Alzheimer, is the primary cause of dementia in the elderly population worldwide [1,2].

Recent statistics show that about 55 million people around the world suffer from AD or another type of dementia [3].

As population aging has been recognized as a universal phenomenon in recent years, and as AD is a strongly age-related disease, the increasing number of AD patients will lead to tremendous consequences, for both society and the economy, in all countries across the world. At the individual level, AD is associated with a shorter life expectancy, functional disability, and institutionalization, whereas the family and society have to sustain significant costs for daily therapy and persistent care [4].

Lately, scientists have focused on various therapies in order to enhance the life quality of those who suffer significantly from neurodegenerative diseases due to the high incidence of AD in recent decades.

Although in recent years, diverse pharmacologic treatments and stem-cell-targeted therapies have been used to slow the progression of AD, none of them have been fully efficient [5].

Currently, the mechanisms of AD mainly focus on the amyloid cascade, tau protein, neuroinflammation, metal ion, and oxidative stress hypotheses [6].

Alzheimer’s disease is characterized by two pathological hallmarks, namely, the extracellular accumulation of amyloid β (Aβ) peptides, generated by the cleavage of amyloid precursor proteins (APPs), and the intracellular accumulation of hyperphosphorylated tau, manifested as neurofibrillary tangles (NFTs) [7].

Nowadays, it is accepted that Aβ aggregation may be considered an essential trigger in AD and may induce tau misfolding, tau-mediated toxicity, accumulation in tangles, and tau spreading, which lead to neuronal dysfunction, neurodegeneration, and dementia [8,9,10,11]. Conversely, Aβ toxicity is also tau-dependent [9].

The accumulation of Aβ and hyperphosphorylated tau proteins, which is an abnormal post-translational modification, could independently exacerbate mitochondrial dysfunction and ROS production [12,13].

It is well known that the brain is vulnerable to ROS due to its intense oxidative metabolism, high levels of polyunsaturated fatty acids, and high number of resident immune cells [14].

Scientific evidence supports the link between Aβ-induced oxidative stress, synaptic dysfunction, and the early onset or progression of AD due to mitochondrial damage, abnormal metal homeostasis, and activated microglia ROS production [12,15].

Additionally, it is well accepted that neuroinflammation is also associated with memory deficits and cognitive impairment in AD patients. Scientific studies have shown an association between the two hallmarks and cognitive decline [8,16].

Furthermore, Aβ may activate the Nuclear factor kappa B (NFkB) signaling pathway (via the extracellular signal-regulated kinase (ERK) and mitogen-activated protein kinase (MAPK) pathways), an important regulator of the generation of inflammatory cytokines [16,17].

Considering the multiple hypotheses regarding the pathogenesis of AD (i.e., inflammation, oxidative stress, mitochondrial dysfunction, altered metal homeostasis, axonal transport deficits, the extracellular accumulation of Aβ peptides, and the intracellular accumulation of hyperphosphorylated tau proteins), there is an urgent need to improve treatment [12,18]. However, neuroprotection may not fully stop disease progression, but the administration of multiple compounds with diverse properties may delay the onset, slow progression, or at least provide some relief of symptoms [19,20].

The *Betula* genus comprises approximately 150 common trees and shrubs, widely spread in temperate regions around the world [21,22]. Vegetal medicinal products are represented by leaves, bark, bugs, juice, and distilled oil [22] and are known for their diuretic, anti-rheumatic, anti-inflammatory, antimicrobial, and analgesic properties [21,22,23,24,25]. Antioxidant and antitumoral activities have also been reported [22,23,26,27]. All these biological activities are attributed to the chemical composition of these species, which include various classes of compounds, such as polyphenols (flavones, lignans, phenols, and tannins) and terpenes (sesquiterpenes and triterpenoids) [21,23,25,27].

*Betula pendula* Roth. (Betulaceae family), commonly known as birch or silver birch, is a large tree, with alternate, deciduous, and serrated leaves; male flowers in pendulous spikes; and female flowers in erect or pendulum spikes [28]. It is one of the most well-known species of the *Betula* genus, traditionally used for various medicinal purposes, such as the treatment of skin disorders, inflammation, rheumatism, and urinary disorders [23,25,26,27].

The Committee of Herbal Medicinal products (EMA, 573240/2014) defines birch leaf as the common name of the leaves of *Betula pendula* Roth and/or *Betula pubescens* Ehrh. or hybrids of both species and describes their use in different formulations (infusions, dry extracts, herbal extracts, powders, and stabilized juices) for their diuretic, anti-rheumatic, and anti-inflammatory properties [29].

The leaves of *B. pendula* have been studied for a large number of other pharmacological properties, such as anti-tyrosinase [22], antioxidant [26], anti-inflammatory [24], antibacterial [27], and gastroprotective [25] activities, and they have demonstrated significant medicinal potential.

Based on these data, our study aimed to evaluate the effects of *B. pendula* leaf extract (BPE) on spatial working memory, general locomotion, lipid peroxidation, inflammatory cytokines, various protein expressions, the NFkB signaling pathway, and histopathological changes in the hippocampus of Aβ_1-42_-treated rats.

## 2. Material and Methods

### 2.1. Reagents

Aβ_1-42_ (Sigma, St. Louis, MO, USA), Malondialdehyde (MDA)-kit Elabscience (Wuhan, China), primary antibodies against pTau (phosphorilated at Ser 404 and Ser 396), Nuclear factor kappa B( NFkB) (Cell Signaling Technology, Danvers, MA, USA) COX 2 and pNFkB (p-RELA/NFκB p65 Antibody, 27.Ser 536) antibodies, GAPDH (Santa Cruz Biotechnology, Santa Cruz, CA, USA), anti-synaptophysin antibodies (Merck Millipore, Darmstadt, Germany), secondary antibodies HRP-conjugated (Cell Signaling Technology and Santa Cruz Biotechnology) were obtained. All the other solvents and reagents used for analysis were of analytical grade purity and were purchased from Merck KgaA (Darmstadt, Germany). The reference compounds used in the LC/MS method were also of high purity and were purchased from Phytolab (Vestenbergsgreuth, Germany).

### 2.2. Vegetal Material

*B. pendula* leaves were collected from the spontaneous flora of Cluj County (Romania), and vegetal material was identified at the Department of Pharmacognosy of the “Iuliu Haţieganu” University of Medicine and Pharmacy, Cluj-Napoca, by Lecturer Irina Ielciu, PhD. Voucher specimens were deposited at the Herbarium of the department (voucher no. 852). The collected vegetal material was subsequently used to obtain the extract to be tested in phytochemical and pharmacological assays.

### 2.3. Extraction Procedure and Sample Preparation

*B. pendula* leaf extract (BPE) was obtained via the maceration of fresh leaves with 50% V/V ethanol in water for 10 days, with 2–3 shakes/day, using a fresh-plant-to-solvent extraction ratio of 1 to 2. Plant humidity was established at 60.03%, while the physicochemical properties of the extract were evaluated at 0.979 for relative density, 4.47% for the dry residue, and 33% *v*/*v* for the ethanolic content, using the methods described by the European Pharmacopoeia. The obtained macerate was filtered through Whatman filter paper no. 1. Prior to in vivo administration, all of the ethanol was removed by using a rotary evaporator (Hahnvapor, HS-2000NS, Hahnshin Scientific Co., Sejong, Republic of Korea) at a temperature of 40 °C, and, subsequently, the sample was reconstituted with distilled water and immediately administered to animals [30,31,32,33].

### 2.4. Spectrophotometric Assays

All spectrophotometric assays were performed on a Cintra 101 spectrophotometer (GBC Scientific Equipment, Dandenong, VIC, Australia).

#### 2.4.1. Total Polyphenolic Content (TPC)

Next, 1 mL of BPE was diluted to 10 mL with methanol; 0.5 mL of phosphotungstic reagent was added to 0.1 mL of this solution. The mixture was adjusted to 25 mL with 15% sodium carbonate solution. The blank solution was prepared by adding 0.1 mL BPE diluted to 25 mL with 15% sodium carbonate solution. For the calibration curve, 0.5 mL of phosphotungstic reagent was added to 0.5 mL of different concentrations of gallic acid (GA) solutions, and then the mixture was adjusted to 25 mL with 15% sodium carbonate solution. Absorbances were measured at 715 nm after 2 min, and the results are expressed as mg gallic acid equivalents (GAE)/100 mL extract [30,34].

#### 2.4.2. Total Flavonoid Content (TFC)

Then, 5 mL of 10% sodium acetate solution and 3 mL of 2.5% aluminum chloride solution were added to 0.5 mL of BPE. Afterwards, the mixture was adjusted to 25 mL with methanol. For the blank solution, 8 mL of water was added to 0.5 mL BPE, and then the mixture was diluted to 25 mL with methanol. For the calibration curve, 5 mL of 10% sodium acetate solution and 3 mL of 2.5% aluminum chloride solutions were added to 0.5 mL of different concentrations of rutoside solutions, and then the mixture was adjusted to 25 mL with methanol. For the standard blank, 8 mL of water was added to 0.5 mL reference solution and diluted to 25 mL with methanol. All absorbances were measured at 430 nm after 30 min, and the results are expressed as mg rutoside equivalents (RE)/100 mL extract [30,34].

### 2.5. LC/MS Analysis

An LC/MS analysis was carried out on a Shimadzu Nexera I LC/MS-8045 (Kyoto, Japan) UHPLC system equipped with a quaternary pump, autosampler, ESI probe, and quadrupole rod mass spectrometer. Separation was achieved on a Luna C18 reversed-phase column (150 mm × 4.6 mm × 3 mm, 100 Å, Phenomenex—Torrance, CA, USA). The column temperature was maintained at 40° C degrees during the analysis. The mobile phase consisted of a gradient of LC/MS-grade methanol (Merck, Darmstadt, Germany) and ultrapurified water obtained using a Simplicity Ultra-Pure Water Purification System (Merck Millipore, Billerica, MA, USA). The organic modifier used for analysis was LC/MS-grade formic acid (Merck, Darmstadt, Germany). The flow rate was maintained at 0.5 mL/min. The total analysis time was 35 min.

Detection was performed on a quadrupole rod mass spectrometer operated with electrospray ionization (ESI) in negative and positive multiple reaction monitoring (MRM) ion mode. The interface temperature was set to 300 °C degrees. Nitrogen at 35 psi and 10 L/min was used as a drying gas. The capillary potential was set to +3000 V [31,32,33].

### 2.6. Antioxidant Capacity Assays

#### 2.6.1. The DPPH Method

The DPPH method is based on the change in color of the 2,2-diphenyl-1-picrylhydrazyl free radical (violet color), which, in the presence of an antioxidant, is reduced, and the color turns to yellow. In this way, the color change can be correlated with the antioxidant capacity using a spectrophotometric measurement. For the assay, 1 mL of BPE was diluted to 10 mL with methanol. Then, 0.3 mL, 0.6 mL, and 0.9 mL of this solution were further diluted to 10 mL with methanol to obtain samples with different antioxidant concentrations. Next, 25 mM DPPH was added to 5 mL of these dilutions, and these mixtures were incubated for 30 min at 40° C. In the same manner, a reference solution was prepared using 5 mL of 25 mM DPPH solution and 5 mL methanol. Methanol was used as a blank. Spectrophotometric determination was performed at 517 nm. The free DPPH radical inhibition percentages were determined for each antioxidant concentration, while IC_50_ values were determined from the curves built for each sample (concentration as function of inhibition percentage). The following formula was used to determine the inhibition percentage: %I = (Ar − As) × 100/Ar, where Ar represents the absorbance of the reference solution, and As represents the absorbance of the solutions with samples [34,35,36,37].

#### 2.6.2. FRAP Method

This method is based on the change in the color of an iron complex with the 2,4,6-tripyridyl-s-triazine radical (TPTZ) and, more specifically, on the reduction of ferric ion to ferrous iron in this complex. The color changes from a light yellowish green to blue and can be easily correlated with the antioxidant power via spectrophotometric determination. To 2.5 mL 10 mM TPTZ solution in 40 mM hydrochloric acid, 2.5 mL 20 mM ferric chloride solution and 25 mL acetate buffer (pH = 3.6) were added. In this way, the FRAP reagent was prepared. Subsequently, to 0.01 mL BPE, water was added to reach 0.8 mL, and then 6 mL FRAP reagent was added. In the same manner, a blank solution using water instead of the sample was prepared. Spectrophotometric determination was performed at 593 nm, and the antioxidant capacity was evaluated by measuring the mM Trolox equivalents (TE)/100 mL extract [34,35,38].

### 2.7. Animals and Experimental Design

Experimental procedures were approved by the Animal Ethics Board of “Iuliu Hatieganu” University on animal welfare according to the Directive 2010/63/EU on the protection of animals used for scientific purposes (Authorization No 325/12.08.2022). Twenty-eight Wistar male rats were used under standard laboratory conditions, housed in a 12 h light–12 h dark cycle at room temperature (24 ± 2 °C). The rats had free access to a standard normocaloric pellet diet and received water ad libitum.

**Figure 1:**
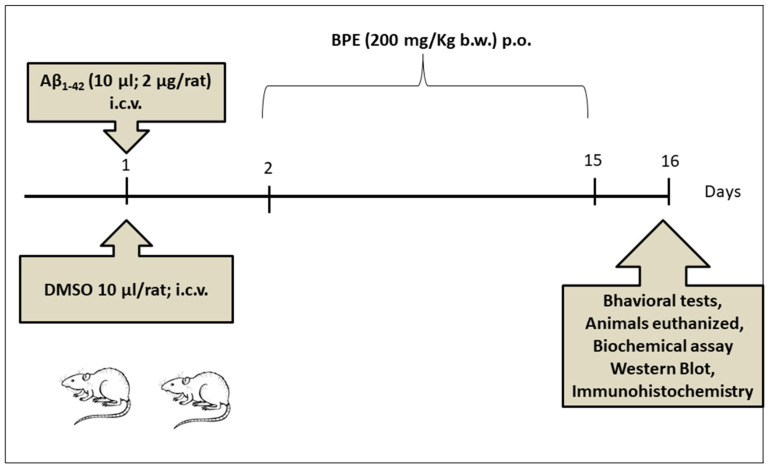
Experimental design: 28 Wistar rats were divided into 4 groups (*n* = 7/group): control (1); untreated animals + drinking water, amyloid (2 μg/rat) (2); drinking water, amyloid + *B. pendula* leaf extract (BPE) (3); and DMSO + drinking water (4) (as amyloid was dissolved in DMDO). On the 1st day, one dose (2 µg/rat) of Aβ_1-42_ was intracerebroventricular (i.c.v.) administered. Subsequently, BPE (200 mg/kg b.w.) was orally administered for the next 14 days. Groups 1, 2, and 4 received drinking water for the next 14 days. On the 16th day of experiment, behavioral tests were performed, and then, under anesthesia with an intraperitoneal injection of ketamine/xylazine cocktail (90 mg/kg b.w. ketamine and 10 mg/kg b.w. xylazine), all animals were euthanized. The blood and hippocampus were harvested for oxidative stress assays and ELISA tests. Additionally, hippocampus fragments were taken for Western blotting, histopathological examination, and immunohistochemistry.

To evaluate the effect of BPE on ambulatory activity, markers of lipid peroxidation, inflammation, the NF-kB signaling pathway, and histopathological changes in the brain of Aβ_1-42_ amyloid-treated rats, the animals were divided into 4 groups of 7 rats each (Figure 1). Group one served as the control. On the first day of the experiment, the animals in groups 2 and 3 received Aβ_1-42_ (2 μg/rat bilaterally;i intracerebroventricular (i.c.v)), and the animals in group 4 received DMSO 1% (10 μL/rat; i.c.v). The animals in group 3 were orally administered BPE (200 mg/kg b.w.) for the next 14 days. On the 16th day of the experiment, the Y-maze test and open-field test (OFT) were conducted; afterwards, all animals were euthanized under anesthesia with an intraperitoneal injection of a ketamine/xylazine cocktail (90 mg/kg b.w. ketamine and 10 mg/kg b.w. xylazine). The blood and hippocampus were collected for oxidative stress assays and ELISA tests. Additionally, hippocampus fragments were taken for Western blotting, routine histopathology, and immunohistochemistry.

An Aβ_1-42_ (Sigma, St. Louis, MO, USA) solution was prepared as described previously by Chromy et al. (2003) [39], Morroni et al. (2016) [40], and Sheng et al. (2017) [41]. Aβ_1-42_ (5 μL × 2) was injected bilaterally into the lateral ventricles through a microinjector with the following coordinates: AP = −4.8 mm from the bregma, ML = ±3.5 mm, and DV = −4 mm from the dura mater, according to the Paxinos and Watson rat brain atlas for stereotaxic surgery [42]. For the sham group, an equivalent volume of DMSO was injected bilaterally into the lateral ventricles through a Hamilton syringe, using the above-mentioned coordinates.

### 2.8. Behavioral Testing

A test widely used to evaluate general locomotion and emotionality—like behavior—in rodents is the open-field test (OFT). A visual tracking system (Smart Basic Software version 3.0 Panlab Harvard Apparatus), using specific mazes for rats (Ugo Basil Animal Mazes for Video-Tracking, 40172, 47100), recorded the animals’ continuous behavior for a 5 min period. In OFT, activities such as the total and peripheral travelled distance and the number of entries are indicators of general locomotor activity. A high center travelled distance and number of entries and a high center time ratio (center/total time) are reported as low emotionality-related indicators [43,44,45].

The Y-maze test assesses spatial working memory, which is a form of short-term memory. Smart Software (Smart v.3.0.06) scored the spontaneous alternations in the three arms of the maze, based on the animals’ free exploratory behavior. Consecutive entries into each arm of the maze, without repeated entries, are considered an alternation, and this is expressed as a percentage of the total arm entries. In order to remove residual odor, the mazes were cleaned with 70% ethanol between tasks [46,47].

### 2.9. Biochemical Investigations of Oxidative Stress

Malondialdehyde (MDA) levels were studied as end-products of the peroxidation of polyunsaturated fatty acids. Peroxidase (PRx), catalase (CAT), and superoxide dismutase (SOD) activities were evaluated as enzymatic antioxidant activities. The MDA levels and PRx, CAT, and SOD activities in the plasma and hippocampus were determined as described by Conti [48], Pippenger [49], and Fridovich [50].

### 2.10. Quantitative Estimation of Tumor Necrosis Factor-α (TNF-α) and Interleukin 1β (IL-1β) Levels Using ELISA Technique

The concentrations of proinflammatory cytokines (such as TNF-α and IL-1β) were determined using the ELISA technique (Rat Inflammation ELISA Strip for Profiling Cytokines) according to the manufacturer’s protocol (Elabscience). The results are expressed as pg/mg protein with associated standard deviations.

### 2.11. Evaluation of Phospho–Tau (Ser 404 and S396), Synaptophysin, Cyclooxygenase-2 (COX 2), NFkB, and pNFkB Protein Expressions

Evaluations of the protein levels of pTau Ser404 and pTau Ser396 (phosphorylated tau at Ser 404 and 396, respectively), synaptophysin, COX 2, NFkB, and pNFkB (p-RELA/NFkB p65) were carried out through Western blot; 20 μg of protein/sample from tissue lysates were used. Samples were run on 12% SDS-PAGE gel via electrophoresis using a Biorad Miniprotean system and then transferred to polyvinylidenedifluoride membranes using a Trans Blot Turbo^TM^ Transfer System (BioRad, Hercules, CA, USA). The primary antibodies used were anti-pTau Ser396, anti-pTau Ser404, anti-synaptophysin, anti-COX 2, anti-NFkB, and anti-pNFkB. Membranes were then washed and incubated with the corresponding secondary HRP-linked antibodies. Bands were detected via chemoluminiscence, using a Thermo Scientific™ SuperSignal™ West Femto Maximum Sensitivity Substrate (Thermo Fisher Scientific, Rockford, IL, USA), a ChemiDoc MP Imaging System, and Image Lab Software (version 6.0.0 build 25, Standard Edition) (Biorad). GAPDH was used to normalize the protein loading of the samples [51].

### 2.12. Histological and Immunohistochemical Investigation of the Brain

At the end of the experiment, the rats were euthanized. A brain histological analysis was performed with 10% buffered formalin-fixed and paraffin-embedded samples, sectioned at 5 μm. For histopathological and immunohistochemical analyses, we examined the global right hippocampal structures. The histological exam was performed with thioflavin S (Sigma Chemical Co., St. Louis, MO, USA), which labeled abnormal beta sheets of the cellular proteins with high confidence for beta-amyloid structures. The sections were deparaffinized and hydrated with distilled water and incubated with an aqueous solution of thioflavin S for 2 h at room temperature. A strong green fluorescence signal of the light-excited thioflavin S was observed on a confocal microscope. For a quantitative analysis, the percentage of positive cells related to various section areas was calculated using ImageJ analysis software. (ImageJ.5.0)

Fixed and paraffin-embedded brain samples were used for the immunohistochemical detection of the phosphorylated tau (p-Tau) at sites Ser(404) (pTau Ser 404). Enzyme-based antigen retrieval in citrate buffer pH 6 was followed by peroxidase blocking with 3% hydrogen peroxide, and then the sections were washed with Tween 20/Tris-buffered saline (TTBS). Non-specific background staining was blocked with 10% bovine serum albumin (BSA) in TTBS pH 7.8 for 2 h. The sections were then incubated overnight at 4 °C with a monoclonal anti-p-Tau antibody (1:500, Cell Signaling Technology, Regional Office, The Nederlands, 2316 WZ Leiden), and then the sections were washed with TTBS and treated with biotinylated–HRP secondary antibody universal link (Dako, LSAB + System-HRP) at room temperature for 30 min. All sections were washed in TTBS and incubated for 30 min with streptavidin–peroxidase (Dako, LSAB+), and then, after being washed three times with TTBS, the sections were incubated with 3, 3′-diaminobenzidine reagent (DAB + Chromogen, Dako, LSAB + System-HRP) for 5 min. To check the specificity of the immunohistochemistry tests, tissues in which primary antibodies were omitted from the initial incubation were also prepared.

Immunohistochemical reactions were scored according to a previous method (Toma et al., 2017) as follows: 0, negative reaction; 0.5–1+, low reaction; 2+, moderate reaction; 3+, intense reaction; and 4+, very intense reaction [52,53,54,55].

### 2.13. Statistical Analysis

A one-way analysis of variance (ANOVA) followed by either Tukey’s post hoc test, to test statistical significance among four groups, or Bonferonni’s post hoc test, to test statistical significance among two groups, was performed using GraphPad Prism software, version 6.0, GraphPad, San Diego, CA, USA. Data are expressed as the mean ± standard deviation (SD). A *p* value lower than 0.05 was considered statistically significant.

## 3. Results

### 3.1. TPC and TFC

The results obtained for the TPC and TFC quantification of BPE are shown in Table 1.

### 3.2. LC/MS Analysis of BPE

The results obtained for the LC/MS analysis of BPE are presented in Table 2.

### 3.3. Antioxidant Assays

The assessment of the antioxidant capacity was performed using a DPPH bleaching assay and the FRAP method. The results are shown in Table 3.

### 3.4. Behavioral Studies

**Figure 2:**
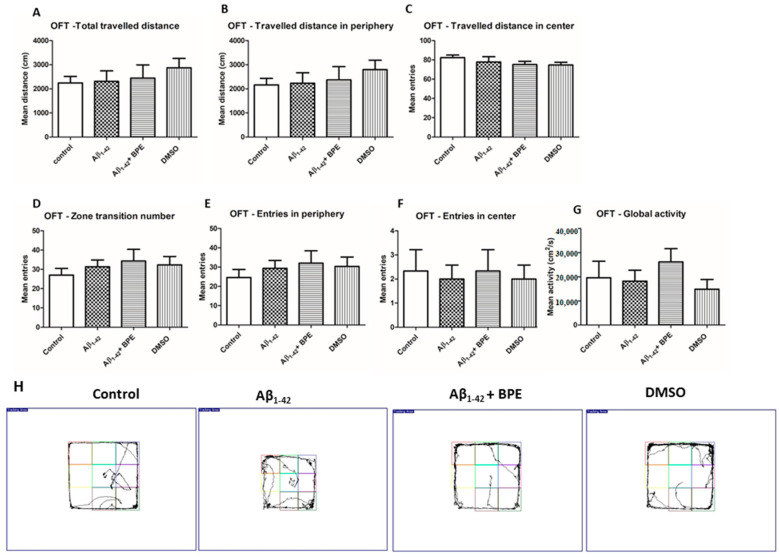
The effects of BPE administration on general locomotion and emotionality—like behavior in open-field test (OFT). BPE administration tended to improve general locomotion and emotionality, like behavior, but without any statistical significance (*p* > 0.05) (**A**–**H**). Each group consisted of 7 rats. The effects of BPE administration on spontaneous alternations in the Y-maze test are illustrated in Figure 3. Spontaneous alternation behavior tests measure the spatial working memory of rodents, which is a form of short-term memory; therefore, we evaluated the effects of BPE on the memory deficits induced by the Aβ_1-42_ injection. Thus, the Aβ_1-42_ group exhibited a significantly higher number of errors in the Y-maze test than the control group (*p* < 0.05). Conversely, BPE administration significantly reversed the impairment of spontaneous alternation behavior (*p* < 0.001).

The effects of BPE administration on general locomotion and emotionality—like behavior in the open-field test (OFT)—are illustrated in Figure 2. BPE administration tended to improve general locomotion, but without statistical significance (*p* > 0.05).

### 3.5. Oxidative Stress Assessment in Plasma and Brain

**Figure 4:**
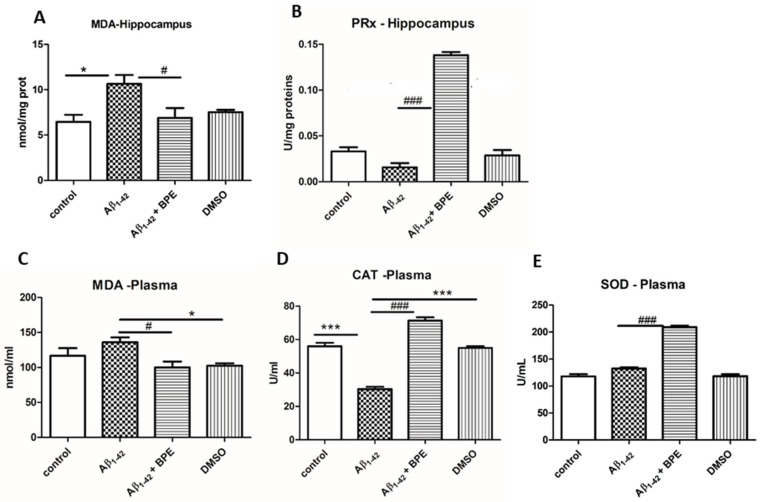
The effects of BPE administration on malondialdehyde (MDA) levels (**A**,**C**) and on peroxidase (PRx) (**B**), catalase (CAT) (**D**), and superoxide dismutase (SOD) (**E**) activities in the hippocampus and plasma of rats. BPE administration decreased lipid peroxidation in the hippocampus and plasma (Aβ_1-42_ + BPE vs. Aβ_1-42_, *p* < 0.05) (**A**,**C**). Significantly higher levels of MDA were recorded in the hippocampus and plasma of the Aβ_1-42_-treated group (Aβ_1-42_ vs. control, *p* < 0.05) (**A**); Aβ_1-42_ vs. DMSO, *p* < 0.05 (**C**). In the hippocampus, PRx displayed higher activity after BPE treatment (Aβ_1-42_ + BPE vs. Aβ_1-42_, *p* < 0.001) (**B**). In the plasma, both CAT and SOD increased after BPE treatment (Aβ_1-42_ + BPE vs. Aβ_1-42_, *p* < 0.001) (**D**,**E**). CAT displayed lower levels than either control or DMSO group after Aβ_1-42_ treatment (Aβ_1-42_ vs. control/DMSO, *p* < 0.001) (**D**). Each group consisted of 7 rats. Aβ_1-42_ vs. control/DMSO, *; Aβ_1-42_ vs. Aβ_1-42_ + BPE, #. Results are expressed as mean ± SD; #,*, *p* < 0.05; ###, ***, *p* < 0.001.

The malondialdehyde (MDA) levels, catalase (CAT), superoxide dismutase (SOD) and peroxidase (PRx) activities in the hippocampus and plasma of the rats treated with BPE after Aβ_1-42_ administration are illustrated in Figure 4. BPE administration decreased lipid peroxidation in the hippocampus and plasma (Aβ_1-42_ + BPE vs. Aβ_1-42_, *p* < 0.05). Significantly higher levels of MDA were recorded in the hippocampus and plasma of the Aβ_1-42_-treated group (Aβ_1-42_ vs. control, *p* < 0.05; Aβ_1-42_ vs. DMSO, *p* < 0.05). In plasma, both CAT and SOD activities increased after BPE treatment (Aβ_1-42_ + BPE vs. Aβ_1-42_, *p* < 0.001). CAT displayed lower levels than either the control or DMSO group after Aβ_1-42_ treatment (Aβ_1-42_ vs. control/DMSO, *p* < 0.001). In the hippocampus, PRx displayed higher activity after BPE treatment (Aβ_1-42_ + BPE vs. Aβ_1-42_, *p* < 0.001).

### 3.6. Quantitative Estimation of TNF-α and IL-1β Levels Using ELISA Technique

**Figure 5:**
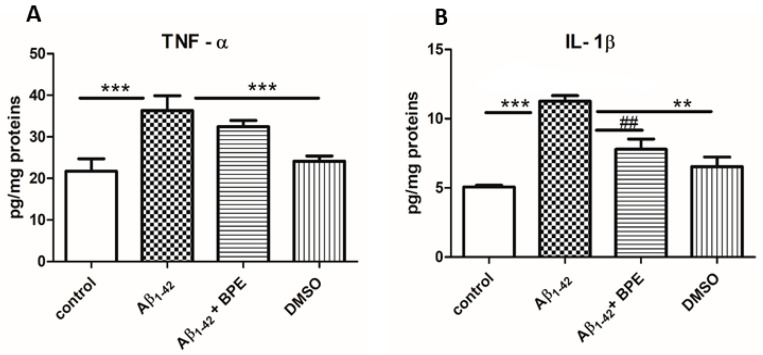
The effects of BPE administration on tumor necrosis factor-α (TNF-α) and Interleukin 1β (IL-1β) levels in the hippocampus of rats (**A**,**B**). Significantly increased levels of TNF-α (**A**) and IL-1β (**B**) were observed in the hippocampus of Aβ_1-42_-treated rats (Aβ_1-42_ vs. control/DMSO, *p* < 0.05); BPE administration decreased IL-1β levels (Aβ_1-42_ + BPE vs. Aβ_1-42_, *p* < 0.01) (**B**). Each group consisted of 7 rats. Aβ_1-42_ vs. control/DMSO, *; Aβ_1-42_ vs. Aβ_1-42_ + BPE, #. Results are expressed as mean ± SD; ##, **, *p* < 0.01; ***, *p* < 0.001.

The proinflammatory cytokine levels in the hippocampus of the rats treated with BPE after Aβ_1-42_ administration are illustrated in Figure 5. Significantly increased levels of inflammatory cytokines were observed in the hippocampus of Aβ_1-42_-treated rats (*p* < 0.05) as compared to the control and DMSO groups. The hippocampus IL-1β levels were decreased in the rats with BPE treatment (*p* < 0.01).

### 3.7. Evaluation of Tau, Synaptophysin, COX 2, NFkB, and pNFkB Protein Expressions

**Figure 6:**
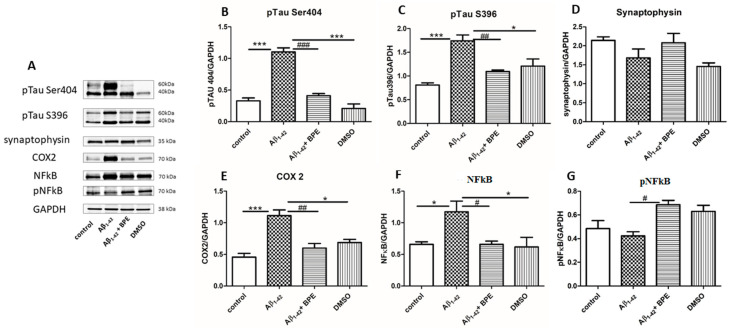
The effects of BPE administration on the expression of Phospho-Tau (Ser396) (pTau Ser 396), Phospho-Tau (Ser404) (pTau Ser404) synaptophysin, cyclooxygenase-2 (COX 2), Nuclear factor kappa B (NFkB), and Phospho NFkB (pNFkB) in the hippocampus (**A**–**G**). Protein expressions were analyzed using Western blot (WB). Image analysis of Western blot band was carried out using densitometry; the results were normalized to GAPDH. Aβ_1-42_ vs. control/DMSO, *; Aβ_1-42_ vs. Aβ_1-42_ + BPE, #. Results are expressed as mean (*n* = 3) ± SD; #,*, *p* < 0.05; ##, *p* < 0.01; ###, ***, *p* < 0.001.

The effects of BPE administration on the expression of pTau Ser396, pTau Ser404 synaptophysin, COX 2, NFkB, and pNFkB in the hippocampus are illustrated in Figure 6. Aβ_1-42_ significantly upregulated the expressions of the phosphorylated tau protein (pTau) Ser404 (Aβ_1-42_ vs. control/DMSO, *p* < 0.001) and pTau S396 (Aβ_1-42_ vs. control, *p* < 0.001; Aβ_1-42_ vs. DMSO, *p* < 0.05) in the hippocampus of the rats, as compared with the control group. BPE treatment reduced the levels of pTau Ser404 and pTau S396 (*p* < 0.001) in the hippocampus as compared to the Aβ_1-42_-treated group. Synaptophysin levels increased after BPE treatment (Aβ_1-42_ + BPE: 2.078 ± 0.43 vs. Aβ_1-42_: 1.682 ± 0.40 *p* > 0.05), but the differences were not statistically significant. In the hippocampus, a significantly higher expression of COX 2 was recorded in the Aβ_1-42_ group than in the control and DMSO groups (Aβ_1-42_ vs. control, *p* < 0.001; Aβ_1-42_ vs. DMSO, *p* < 0.05); BPE downregulated the expression of COX 2 (Aβ_1-42_ + BPE vs. Aβ_1-42_, *p* <0.01). Aβ_1-42_ significantly stimulated the total level of NFkB in the hippocampus (Aβ_1-42_ vs. control, *p* < 0.05; Aβ_1-42_ vs. DMSO, *p* < 0.05); BPE downregulated the expression of total NFkB (Aβ_1-42_ + BPE vs. Aβ_1-42_, *p* < 0.05); BPE increased the expression of pNFkB (the active form) as compared to Aβ_1-42_ (*p* < 0.05).

### 3.8. Histological and Immunohistochemical Investigation of the Hippocampal Formation

**Figure 7:**
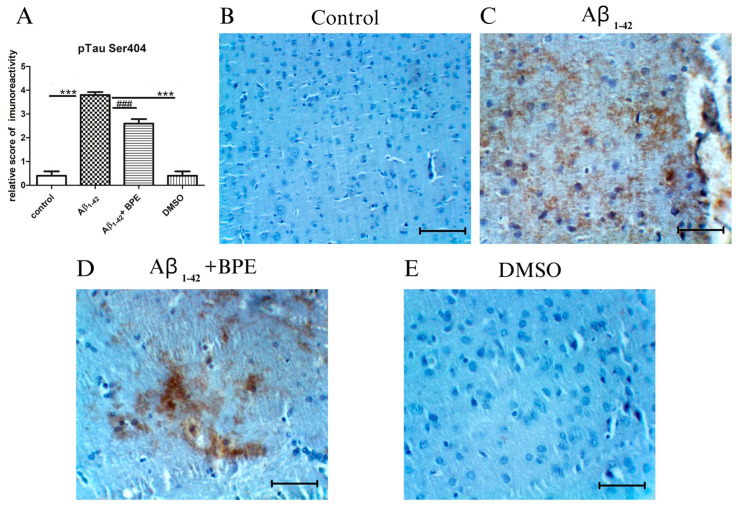
The effects of BPE administration on the immunoreactivity of tau phosphorylation in the hippocampal formation ×200. Scale bar = 50 µm. Phospho-Tau Ser404 showed strong immunoreactivity in the hippocampal formation of Aβ_1-42_-treated rats as compared to other groups (Aβ_1-42_ vs. control/DMSO, *p* < 0.001). BPE administration diminished the hyperphosphorylation of tau proteins (Aβ_1-42_ + BPE vs. Aβ_1-42_, *p* < 0.001).Aβ_1-42_ vs. control/DMSO, *; Aβ_1-42_ vs. Aβ_1-42_ + BPE, #. Results are expressed as mean ± SD; ###, ***, *p* < 0.001.

The immunoreactivity of tau phosphorylation in the hippocampal formation of the rats treated with BPE after Aβ_1-42_ administration is illustrated in Figure 7. Phospho-Tau Ser404 showed a strong immunoreactivity in the hippocampal formation of Aβ_1-42_-treated rats as compared to the other groups (Aβ_1-42_ vs. control/DMSO, *p* < 0.001). BPE administration diminished the hyperphosphorylation of tau proteins (Aβ_1-42_ + BPE vs. Aβ_1-42_, *p* < 0.001) (Figure 7A).

**Figure 8:**
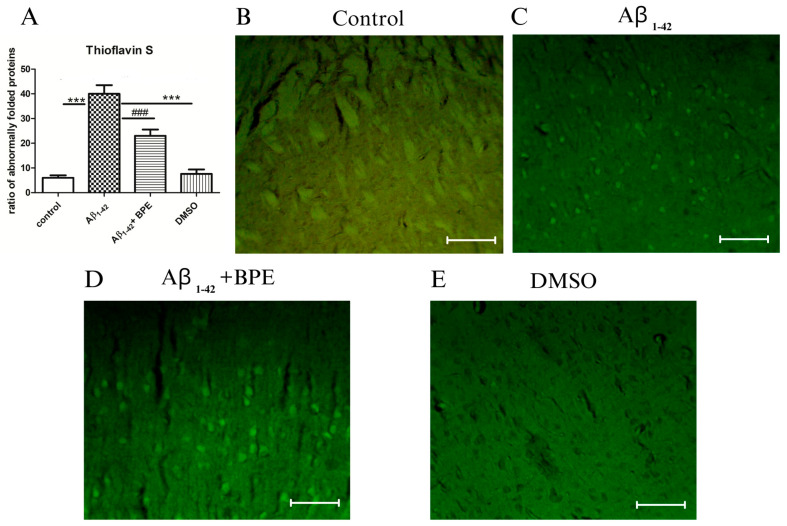
The effects of BPE administration on the misfolded proteins evidenced by thioflavin S staining in the hippocampal formation ×200. Scale bar = 50 µm. A significant accumulation of misfolded proteins was noticed after thioflavin S staining in the Aβ_1-42_-treated rats compared to the control (Aβ_1-42_ vs. control/DMSO, *p* < 0.001) and treated groups (Aβ_1-42_ vs. Aβ_1-42_ + BPE, *p* < 0.001). Aβ_1-42_ vs. control/DMSO, *; Aβ_1-42_ vs. Aβ_1-42_ + BPE, #. Results are expressed as mean ± SD; ###, ***, *p* < 0.001.

The thioflavin S staining in the hippocampal formation of the rats treated with BPE after Aβ_1-42_ administration is depicted in Figure 8. A significant accumulation of misfolded proteins was noticed after thioflavin S staining in the Aβ_1-42_-treated rats compared to the control (Aβ_1-42_ vs. control/DMSO, *p* < 0.001) and treated groups (Aβ_1-42_ vs. Aβ_1-42_ + BPE, *p* < 0.001) (Figure 8A).

## 4. Discussion

In the current study, we evaluated the effects of BPE on general locomotion, spatial working memory, hippocampus oxidative stress (MDA, PRx, CAT, and SOD), inflammation (cytokines: TNF-α, IL-1β, and COX 2), the expression of various proteins (pTau Ser 404, pTau Ser 396, synaptophysin, and the NFkB signaling pathway), and histological aspects in rats following Aβ_1-42_ i.c.v. administration. Our results demonstrate that BPE diminished lipid peroxidation, diminished neuroinflammation, modulated specific protein expression, enhanced antioxidant capacity, and improved spontaneous alternation behavior, suggesting that it has beneficial effects in AD.

The animal model of this study was chosen because it reflects the pathogenetic mechanism of the disease. It is suggested that the accumulation of toxic Aβ proteins in the central nervous system (CNS), due to an imbalance between Aβ production and its clearance, may be the main cause of Alzheimer’s disease. Therefore, as a consequence of Aβ plaque formation in brain tissue, an i.c.v. injection of Aβ molecules has been commonly used as an animal model of AD, as it may eventually lead to cell dysfunction and death [56,57,58].

Although the mechanisms leading to neurodegeneration are multifactorial, such as genetic, environmental, and endogenous factors (brain aging), oxidative stress production and inflammation are considered to play significant roles in neurodegenerative disorders. Furthermore, as brain membrane phospholipids are composed of polyunsaturated fatty acids, brain tissue is highly susceptible to oxidative stress, as it can cause cellular damage and important functional alterations.

There is compelling evidence that provides support for the involvement of oxidative stress in neurodegenerative disorders, including AD [59].

Reactive oxygen species (ROS)-induced cellular damage and the diminished activity of superoxide dismutases (SODs), catalases (CATs), and peroxiredoxins (Prxs), along with activated glial cells, which express iNOS, COX-2, and NADPH oxidase and myeloperoxidase, may contribute to oxidative-stress-mediated neurodegeneration. Moreover, it is considered that inflammatory mediators (tumor necrosis factors (TNF-α); nitric oxide (NO); inducible nitric oxide synthase (iNOS); nicotinamide adenine dinucleotide phosphate (NADPH) oxidase; and cytokines, such as interleukin-1 (IL-1β)) are responsible for oxidative-stress-mediated inflammation [60,61,62].

Additionally, it has been observed that oxidative stress may occur early in the course of AD, thus playing a key role in AD pathogenesis. Therefore, *B. pendula* leaf extract (BPE) was chosen to be studied in this experimental model due to its chemical compounds and potential beneficial effects. Cholorogenic acid and quercetin in its composition demonstrated protective properties in AD via apoptosis induction, antioxidant effects, and the downregulation of the NF-kB and MAPKs pathways [63,64,65].

Species of the *Betula* genus have traditionally been used as medicine in different parts of the world, as they exhibit various in vitro and in vivo pharmacological activities [28].

In the present study, a hydroalcoholic extract obtained from the leaves of *B. pendula* species was tested on a model of Aβ_1-42_ i.c.v. administration. As a possible mechanism, antioxidant capacity was also tested. The novelty of the present study lies in the fact that, despite all the beneficial effects of these species, the neuroprotective, neurochemical, and behavioral effects of *B. pendula* on Aβ_1-42_ i.c.v. administration have never been studied before.

TPC and TFC showed important amounts of compounds that could be related to the antioxidant activity of BPE. These amounts were significantly higher than the ones previously identified in the leaves of the species [23,26].

Concerning the quantification of individual compounds, high amounts of polyphenolic compounds represented by polyphenolcarboxylic acids (caffeic, chlorogenic, trans-*p*-coumaric, ferulic, gallic, and salicylic acids), as well as flavonoids (luteolin, luteolin-7-O-glucoside, quercetin, hyperoside, and quercitrin), were found in the composition of BPE. These compounds were previously identified and quantified in the composition of the leaves of the species [22,24,25,26,28,66,67].

The chemical composition of the tested extract may be correlated with its antioxidant capacity. In order to evaluate this activity, the DPPH and FRAP methods were used. The results show a significant antioxidant capacity for both assays, with values superior to the ones previously reported [22,23,26]. As a potential mechanism of the antioxidant effect, *Betula* sp. extracts may inhibit lipid peroxidation, stimulate DPPH radical scavenging activity, and increase the activities of several cellular antioxidant enzymes (e.g., SOD, CAT, and GPX) [68,69].

The results of the present study show that 14 days of BPE administration diminished lipid peroxidation in the hippocampus and plasma and improved antioxidant activity in the plasma of the Aβ_1-42_ treated rats. These observations confirm that impaired antioxidant activity and increased oxidative stress in brain tissue are involved in the etiology of neurodegenerative disorders [13].

It is noteworthy that chlorogenic acid, ferulic acid, p-coumaric acid, or quercetin may exert neuroprotection against Aβ accumulation by improving neural growth factors and endogenous antioxidant defense mechanisms, decreasing lipid peroxidation, attenuating neuroinflammation, and downregulating NF-kB pathways [63,64,65]. To interfere with the mechanisms involved in AD, can recommend the use of these compounds in neurodegenerative diseases, including AD.

Moreover, as oxidative stress and inflammation are closely related, and one can easily be induced by the other, the levels of proinflammatory cytokines (TNF-α and IL-1β) and COX 2 expression were examined. In the present research, we provide evidence that the Aβ_1-42_-induced rise in IL-1β secretion and COX 2 expression in the hippocampus was prevented by BPE administration. So, indeed, we reached similar conclusions to those mentioned in the literature regarding the anti-inflammatory effect of *B. pendula* extract. As reported previously, the mechanism underlying the anti-inflammatory effect of *Betula* species is related to the inhibition of PGE2 and COX-2; the inhibition of the growth and cell division of inflammatory lymphocytes; the selective induction of the apoptosis of activated T cells; the reduction in the levels of inflammatory mediators, such as IL-6, TNF-α, MMP-1, MMP13, and PGE2; and the release of nitrites, iNOS, and NF-κB in various tissues [24,70].

Another two major hallmarks characteristic of AD are the accumulation of extracellular Aβ plaques and the hyperphosphorylation of tau, leading to the formation of intraneuronal neurofibrillary tangles (NFTs).

Tau is a major microtubule-associated protein (MAP) of a mature neuron, known for its ability to promote assembly and to maintain the structure of microtubules [71]. The accumulation of hyperphosphorylated tau causes impaired synaptic plasticity, neuronal dysfunction, and the formation of neurofibrillary tangles. At the synaptic level, hypephosphorylated tau may either disrupt vesicle release or cause dendritic loss with aberrant postsynaptic activity and cognitive dysfunction [72,73].

Many studies have consistently reported that the hyperphosphorylation of tau at several residues (serine, threonine, or tyrosine) may occur at various stages of disease progression, but the phosphorylation of tau proteins at sites Ser 396–404 is considered, by some authors, to be one of the earliest events in Alzheimer’s disease. Moreover, pTau-S396 was found to be strongly associated with Ab plaques [7,74,75,76].

Consequently, we evaluated the expression of pTau Ser404 and pTau S396 in the hippocampus. Our study confirmed that BPE administration in the rats treated with Aβ_1-42_ diminished the expression of pTau Ser 404 and pTau Ser 396 in the hippocampus and, thus, improved neuronal dysfunction.

The results of other studies are consistent with our results. Santacruz et al. (2005) [77] revealed that cognitive deficiencies may correlate with the appearance of soluble hyperphosphorylated tau. ROS, Aβ, and pTau may influence *N*-methyl-D-aspartate (NMDA) receptors, an ionotropic family of glutamate receptors, which, along with α-amino-3-hydroxy-5-methyl-4-isoxa-zolepropionic acid (AMPA) receptors, may regulate excitatory synaptic transmission and plasticity in the brain, thus modulating learning and memory [78]. Therefore, spontaneous alternation behavior was recorded as a measure of spatial working memory, which is a form of short-term memory. Regarding the behavioral tests, our findings show that BPE treatment enhanced the spontaneous alternation behavior of the Aβ_1-42_-treated group in the Y-maze test. So, a possible explanation for the beneficial effects of BPE on cognitive behavior could be the antioxidant and anti-inflammatory effects of this compound, as it could reverse the effects of the Aβ-induced inflammatory response and excessive oxidative stress. Additionally, the downregulation of pTau proteins under BPE can improve the alteration of synaptic plasticity and synaptic dysfunction. Hence, our data are in agreement with those in previous studies that showed the efficacy of plant polyphenols on cognitive processes in AD [79].

In OFT, there were no significant variations in ambulatory activity between the experimental groups.

In this respect, a decline in spatial memory has been identified as one of the earliest behavioral AD manifestations. As spatial memory networks encompass interacting cell populations within the hippocampal formation and interacting cortical regions, we histologically examined the hippocampal formation [80,81,82].

Our data showed more accumulation of misfolded proteins in the thioflavin S staining in the Aβ_1-42_ group than in the treatment group.

As synaptic loss and dysfunction are correlated with reduced cognitive function [83], we also assessed the expression of synaptophysin in the hippocampus. Synaptophysin is a membrane protein localized to synaptic vesicles. Some authors reported reduced synaptophysin concentrations in certain brain areas of AD, data that are contradictory to ours. Although, in our study, the expression of synaptophysin tended to be downregulated in Aβ_1-42_, the differences were not statistically significant. After BPE administration, the increase was not significant either [84]. These contradictory findings can be partially explained by the link between the severity of symptoms and the loss of synaptic density.

As mentioned previously, neuroinflammation, oxidative stress, and apoptosis are major pathological factors involved in AD pathogenesis. The histological characteristics (e.g., Aβ plaque formation and neurofibrillary tangles) may be exacerbated by inflammation, thus promoting neuronal loss and instability, with NFkB being the core of neurodegeneration.

The transcription factor NFkB, constitutively expressed in the central nervous system (CNS), can be activated by several stimuli, such as ROS, IL-1β, TNFα, opioids, β-amyloid, sAPP (secreted amyloid precursor protein), bacterial lipopolysaccharides (LPSs), isoproterenol, ionizing radiation growth factors, and synaptic transmission (glutamate) [85].

The nuclear factor κB (NFkB), a family of five transcription factors, involved in various cellular processes, is known for its role in the inflammatory response, which is highly linked to neurodegeneration. The activation of the NFkB signaling pathway by the overexpression of Toll-like receptors (TLRs), both on microglia and neurons, leads to the release of cytokines and chemokines, thus resulting in the chronic inflammation observed in AD [86,87,88]. Further, the NFkB family also modulates oxidative stress. On this basis, in vitro studies showed increased oxidative stress mediated by the NFkB response to neurons exposed to Aβ, sustained by lipid peroxides and neurodegeneration. Moreover, many studies confirm that Aβ peptides stimulate NFkB gene expression and its nuclear translocation in neurons and glial cells [88,89,90].

Conversely, the NFkB signaling pathway is also involved in normal brain functioning, such as synaptic plasticity, learning, and memory [91].

Therefore, we evaluated the NFkB and pNFkB expressions in the hippocampus in correlation with oxidative stress, inflammation, and the expression of pTau proteins. In our study, Aβ_1-42_ increased the NFkB level in the hippocampus. BPE administration exerted inhibitory effects on NFkB and increased the expression of pNFkB in the hippocampus of the Aβ_1-42_-treated rats. The role of NFkB in neuroprotection and neurodegenration is more complex and sometimes dual depending on the cell type, brain area, and combination of NFkB subunits [92]. Some studies showed a decreased expression of NFkB and BACE1 and, thus, induced Aβ clearance via the curcuminoid treatment of peripheral blood mononuclear cells collected from AD patients. Moreover, resveratrol markedly reduced NFkB signaling stimulated by Aβ in glial cells and reduced neuronal death, and forsythoside B (FTS•B) exerted an anti-NFkB effect and reduced Aβ plaque formation, tau phosphorylation, and microglial activation, leading to improvements in cognitive function [93,94].

### Limitations of the Study

As with the large variety of animal models based on the i.c.v. administration of Aβ_1-42_, the design of the current study is subject to limitations. Although the early behavioral alterations in the rats following the i.c.v. injection of Aβ_1-42_ and the effects of the BPE extract on this experimental model were evaluated, more behavioral tests for the evaluation of learning and memory performance would be helpful. Therefore, in our future experiments, we will consider the administration of several Aβ_1-42_ doses and the evaluation of the animals at various time periods for both short- and long-term memory.

Although studies on animal models of AD have shown promising results, they can only partially mimic human AD. The neuroprotective effect of natural compounds can be limited by their capacity to cross the blood–brain barrier (BBB) and reach the brain. Moreover, considering clinical translation, another limitation of our study may be related to the reduced number of clinical trials on the efficiency of antioxidant compounds in AD pathology. The dose and duration of the treatment may also interfere with the clinical results, in both animal and human pathology. Therefore, further studies are needed to explain the role of natural compounds and their interactions with vital processes in the brain.

## 5. Conclusions

In conclusion, our study demonstrates that BPE extract significantly reversed the impairment of spontaneous alternation behavior; diminished MDA levels; increased CAT and SOD activities; decreased IL-1β levels; downregulated the expression of pTau Ser404, pTau S396, COX 2, and NFkB; and stimulated the expression of pNFkB. Thus, based on these results, we provide evidence for the neuroprotective effects of BPE on Aβ_1-42_-treated rats, at least partially explained by diminished lipid peroxidation, cell signaling, and immune response modulation. BPE also showed a beneficial effect by improving spontaneous alternation behavior.

Our findings indicate that BPE administration might represent a good option in neurodegenerative disorders, exerting beneficial effects by increasing antioxidant defense and counteracting neuroinflammation.

## Figures and Tables

**Figure 3:**
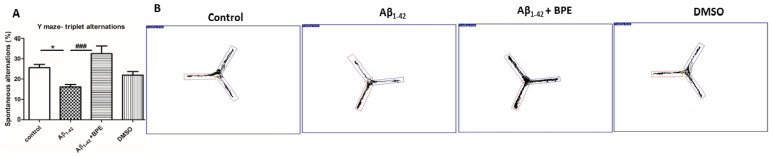
The effects of BPE administration on spontaneous alternations in Y-maze test in rats (**A**,**B**). Aβ_1-42_ group showed a significantly higher number of errors in the Y-maze test than the control group (Aβ_1-42_ vs. control, *p* < 0.05). Conversely, BPE administration increased the percentage of spontaneous alternation behavior (Aβ_1-42_ + BPE vs. Aβ_1-42_, *p* < 0.001). Each group consisted of 7 rats. Aβ_1-42_ vs. control/DMSO, *; Aβ_1-42_ vs. Aβ_1-42_ + BPE, #. Results are expressed as mean ± SD; *, *p* < 0.05; ###, *p* < 0.001.

**Table 1:** TPC and TFC quantification of BPE.

Sample	TPC (g GAE/100 mL Extract)	TFC (g RE/100 mL Extract)
BPE	2.07 ± 0.16	0.73 ± 0.07

**Table 2:** Polyphenolic composition of BPE.

Compound	Retention Time (min)		*m/z* and Main Transition		Concentration (μg/mL)
	Reference	Separated Compound	Reference	Separated Compound	
Caffeic acid	13.8	13.6	179.0 > 135.0	179.0 > 135.0	35 ± 0.003
Chlorogenic acid	12.0	12.0	353.0 > 191.0	353.0 > 191.0	125 ± 0.014
Apigenin	28.2	28.1	269.0 > 117.0	269.0 > 117.0	13 ± 0.001
Chrysin	29.7	30.0	253.0 > 143.0	253.0 > 143.0	1 ± 0.001
Luteolin	26.9	26.8	287.0 > 153.0	287.0 > 153.0	1 ± 0.002
Luteolin-7-O-glucoside	19.9	19.8	447.0 > 284.9	447.0 > 284.9	1 ± 0.05
Quercetin	25.7	27.0	300.9 > 151.0	300.9 > 151.0	40 ± 0.003
Naringenin	26.3	26.9	271.0 > 119.0	271.0 > 119.0	3 ± 0.007
Gallic acid	7.0	7.0	168.9 > 125.0	447.0 > 229.9	72 ± 0.002
Ferulic acid	18.4	18.2	193.0 > 134.0	193.0 > 178.0	20.1 ± 0.01
Trans-p-coumaric acid	17.5	17.9	163.0 > 119.0	163.0 > 93.0	112 ± 0.009
Catechin	10.3	10.3	289.0 > 202.9	289.0 > 202.9	0.4 ± 0.0002
Carnosol	30.6	31.0	329.1 > 285.1	329.1 > 285.1	0.28 ± 0.001
Hyperoside	20.3	20.2	463.1 > 300.0	463.1 > 300.0	2 ± 0.001
Quercetin	25.4	25.1	300.9 > 151.0	300.9 > 121.0	40 ± 0.003
Quercitrin	22.1	22.1	447.0 > 229.9	447.0 > 229.9	99 ± 0.009
Salicylic acid	23.5	23.3	137.0 > 93.0	137.0 > 75.0	14 ± 0.002

**Table 3:** Antioxidant capacity of BPE.

Sample	DPPH IC_50_ (µg/mL)	FRAP µmol TE/100 mL Extract
BPE	31.92 ± 0.0087	9.32 ± 0.0059

## Data Availability

Data are contained within the article. They can be provided upon request by the first author.
